# Genomic characterization of a core set of the USDA-NPGS Ethiopian sorghum germplasm collection: implications for germplasm conservation, evaluation, and utilization in crop improvement

**DOI:** 10.1186/s12864-016-3475-7

**Published:** 2017-01-26

**Authors:** Hugo E. Cuevas, Giseiry Rosa-Valentin, Chad M. Hayes, William L. Rooney, Leo Hoffmann

**Affiliations:** 1 USDA-ARS, Tropical Agriculture Research Station, 2200 Pedro Albizu Campos Avenue, Mayaguez, 00680 Puerto Rico; 2 USDA-ARS, Cropping Systems Research Laboratory, 3810 4th Street, Lubbock, TX 79415 USA; 3 Departments of Soil & Crop Sciences, Texas A&M University, College Station, TX 77843-2474 USA

**Keywords:** *Sorghum bicolor*, Exotic germplasm, Genotyping-by-sequencing, Genome-wide association study, Population structure

## Abstract

**Background:**

The USDA Agriculture Research Service National Plant Germplasm System (NPGS) preserves the largest sorghum germplasm collection in the world, which includes 7,217 accessions from the center of diversity in Ethiopia. The characterization of this exotic germplasm at a genome-wide scale will improve conservation efforts and its utilization in research and breeding programs. Therefore, we phenotyped a representative core set of 374 Ethiopian accessions at two locations for agronomic traits and characterized the genomes.

**Results:**

Using genotyping-by-sequencing, we identified 148,476 single-nucleotide polymorphism (SNP) markers distributed across the entire genome. Over half of the alleles were rare (frequency < 0.05). The genetic profile of each accession was unique (i.e., no duplicates), and the average genetic distance among accessions was 0.70. Based on population structure and cluster analyses, we separated the collection into 11 populations with pairwise *F*_ST_ values ranging from 0.11 to 0.47. In total, 198 accessions (53%) were assigned to one of these populations with an ancestry membership coefficient of larger than 0.60; these covered 90% of the total genomic variation. We characterized these populations based on agronomic and seed compositional traits. We performed a cluster analysis with the sorghum association panel based on 26,026 SNPs and determined that nine of the Ethiopian populations expanded the genetic diversity in the panel. Genome-wide association analysis demonstrated that these low-coverage data and the observed population structure could be employed for the genomic dissection of important phenotypes in this core set of Ethiopian sorghum germplasm.

**Conclusions:**

The NPGS Ethiopian sorghum germplasm is a genetically and phenotypically diverse collection comprising 11 populations with high levels of admixture. Genetic associations with agronomic traits can be used to improve the screening of exotic germplasm for selection of specific populations. We detected many rare alleles, suggesting that this germplasm contains potentially useful undiscovered alleles, but their discovery and characterization will require extensive effort. The genotypic data available for these accessions provide a valuable resource for sorghum breeders and geneticists to effectively improve crops.

**Electronic supplementary material:**

The online version of this article (doi:10.1186/s12864-016-3475-7) contains supplementary material, which is available to authorized users.

## Background

Sorghum [*Sorghum bicolor* (L.) Moench] is the fifth most important grain crop after maize, wheat, rice, and barley [1]. Its role as source of grain and cellulosic-based ethanol is of growing importance. The region of domestication and diversity is located in Northeast Africa, an area that extends from Ethiopia to Sudan [2], but the crop is now grown in an array of environments, ranging from tropical to temperate regions. Sorghum is a highly diverse crop that has experienced multiple re-domestication processes, resulting in five major races differentiated by inflorescence type [3]. These races are associated with particular environments [4], and ongoing evolution in these regions by recombination and selection has led to a highly diverse crop.

Ex situ sorghum germplasm collections (i.e., gene banks) have been established in several countries as a resource for breeding programs. These germplasm collections provide genetic variation for crop improvement programs. Today, the largest worldwide sorghum collection is maintained by the USDA Agriculture Research Service National Plant Germplasm System (NPGS) and includes >41,860 accessions from 114 countries. Likewise, the International Crops Research Institute for the Semi-arid Tropics (ICRISAT), located in India, has a sorghum collection of 37,904 accessions from different countries. The National Genebank of China maintains a sorghum collection of over 16,000 accessions, including 12,000 Chinese sorghum landraces. To use these resources, breeding programs must have an understanding of the variation within the germplasm collection [5]. The establishment of core reference sets (i.e., core collections) that represent the genetic diversity of the broader collection provides a mechanism to classify these resources for the scientific community. In this regard, ICRISAT has developed a core collection of 2,247 accessions based on phenotypic information [6, 7]; this collection was later reduced to a mini-core of 242 accessions based on genetic profiles [8]. A core collection of 2,438 accessions was established for the NPGS sorghum collection using origin site information (i.e., passport information [9]).

The selection of landraces for inclusion in a core set based on passport information alone may not adequately represent the genetic diversity owing to limited passport information for many accessions and/or genetic redundancy (i.e., duplicates). Therefore, the use of a molecular genetics approach is imperative for the conservation and utilization of the collection. However, molecular genetic approaches have been limited to specific samples of sorghum from the NPGS [5, 10–13], and a large portion of the genetic diversity remains uncharacterized in the germplasm collection. These studies confirm that modern sorghum breeding materials are from a narrow genetic base, despite high genetic diversity in the germplasm collection. Recently, a sorghum association panel (SAP) comprising 149 U.S. breeding lines and their 228 progenitor accessions was assembled with the purpose of genetically dissecting economically important traits [14]. Certainly, this panel encompasses a wide range of sorghum genetic diversity that has been exploited in breeding programs, but it is not representative of the vast genetic diversity present in the NPGS collection. Further genomic characterization of a broader range of sorghum diversity is required to both improve conservation efforts and provide new knowledge to promote its integration into sorghum breeding programs.

Genome sequence information and next-generation sequencing technology have made important contributions to the development of new genotyping platforms. Today, genotyping-by-sequencing (GBS) [15] is arguably the most widely used method to genetically characterize plant germplasm. The large number of single-nucleotide polymorphisms (SNPs) identified by GBS makes it possible to integrate into a single research project analyses of genetic diversity and genome-wide association studies (GWAS) [16], and is invaluable for the identification of allelic diversity and rare genetic variation as well as functional analyses. In maize, an analysis of 2,815 inbred accessions from NPGS resulted in the identification of 681,257 SNP markers, half of which were classified as rare variants (frequency < 0.05) [17]. Likewise, in sorghum, an analysis of 971 worldwide sorghum accessions (including the ICRISAT mini-core and SAP) resulted in the identification of ~265,000 SNP markers [16], and an analysis of 1,943 georeferenced sorghum landraces (844 and 1,099 from NPGS and ICRISAT, respectively) was used to identify 404,627 SNP markers [18]. These sorghum genetic diversity studies have substantially improved our understanding of the genetic basis of the domestication process as well as the adaptation of various sorghum races within particular environments. Moreover, they substantiate the use of genotypic and environmental information to predict the phenotypes of accessions. Hence, the phenotypes of accessions within large germplasm collections could be predicted based on prior genotypic and phenotypic analyses of a core subset of accessions.

The Ethiopian germplasm maintained by NPGS contains 7,217 accessions, representing 17% of the sorghum collection [19]. Unfortunately, the geographic coordinates of the collection sites are unknown for 83% of these accessions, limiting the establishment of a representative subset based on passport information alone. An analysis of 137 randomly selected Ethiopian accessions with 20 simple sequence repeat markers revealed abundant genetic diversity divided into two main subpopulations [10]. Four of the five sorghum races and wild forms are present in Ethiopia [20], and previous phenotypic and genotypic analyses identified high levels of genetic diversity in this germplasm [21, 22]. In addition, the geographic distribution of morphological characteristics appears to be determined by topography and climatic variation [21]. Recently, some Ethiopian accessions from the NPGS were genetically characterized for use in GWAS (135 accessions [18]; 31 accessions [16]), but the limited number of accessions and/or the selection approach (i.e., breeding lines and/or ecogeographical regions) may bias estimates of the actual genetic diversity in the collection because these samples were not established to represent the Ethiopian collection. In the current study, we established a core subset of 374 NPGS Ethiopian accessions based on the actual frequency distribution of the known sorghum races in this collection. We phenotyped these accessions for several important agronomic traits at two locations and used GBS to 1) evaluate their genetic and phenotype diversity, 2) determine population structure, 3) study the genetic relationships among accessions (and identify duplicates), 4) determine the genetic relationship between the Ethiopia NPGS collection and the SAP, and 5) explore the potential of this germplasm to elucidate the genetic architecture of quantitative traits using GWAS.

## Results

### GBS of NPGS Ethiopian germplasm

The Ethiopian germplasm core set examined in this experiment comprised 352 exotic accessions and 22 converted tropical lines that are photoperiod-insensitive and dwarf and were produced by crossing exotic lines and modern US cultivars. This core set represented ~5% of the Ethiopian collection, including 16 sorghum races, of which Durra, Durra-bicolor, and unclassified accessions represented 72% of the accessions (Table 1). We pooled tissue from three seedlings for DNA extraction and analyzed the data as a single GBS sample. We obtained approximately 5×sequencing coverage per accession, resulting in the identification of 417,852 SNPs with an average of 34% missing data. We retained SNPs with <20% missing data and minor allele frequencies of >0.01 (i.e., SNPs present in at least three accessions) to obtain a final sample of 148,476 SNPs for further analyses. These SNPs were distributed along the ten sorghum chromosomes with an average of 1 SNP per 5,200 kb; the majority of SNPs were located in sub-telomeric gene-rich regions. In fact, these SNPs tagged 17,196 annotated genes, while another 4,016 genes were within 5 kbp upstream or downstream of a SNP.

**Table 1:** Distribution of sorghum races present in the Ethiopian collection from NPGS

	NPGS^a^ (7,237 accessions)		Representative sample (374 accessions)	
Race	Num. of accessions	%	Num. of accessions^b^	%
Bicolor	90	1.24	6	1.60
Caudatum	145	2.00	8	2.14
Durra-Caudatum	206	2.85	10	2.67
Caudatum-bicolor	78	1.08	5	1.34
Guinea-Caudatum	65	0.90	4	1.07
Kaffir-Caudatum	34	0.47	2	0.53
Mixed	389	5.38	24	6.42
Kaffir-durra	368	5.09	19	5.08
Kaffir-bicolor	24	0.33	2	0.53
Kaffir	47	0.65	2	0.53
Guinea-Kaffir	62	0.86	5	1.34
Guinea-durra	194	2.68	13	3.48
Guinea-bicolor	42	0.58	2	0.53
Guinea	18	0.25	1	0.27
Durra-bicolor	1336	18.47	80	21.39
Durra	2086	28.84	102	27.27
Unknown	2053	28.34	89	23.80

^a^National Plant Germplasm System from United States

^b^Number of randomly selected accession from each race group present in the NPGS Ethiopia collection

### Integrity of NPGS Ethiopian collection

**Fig. 1:**
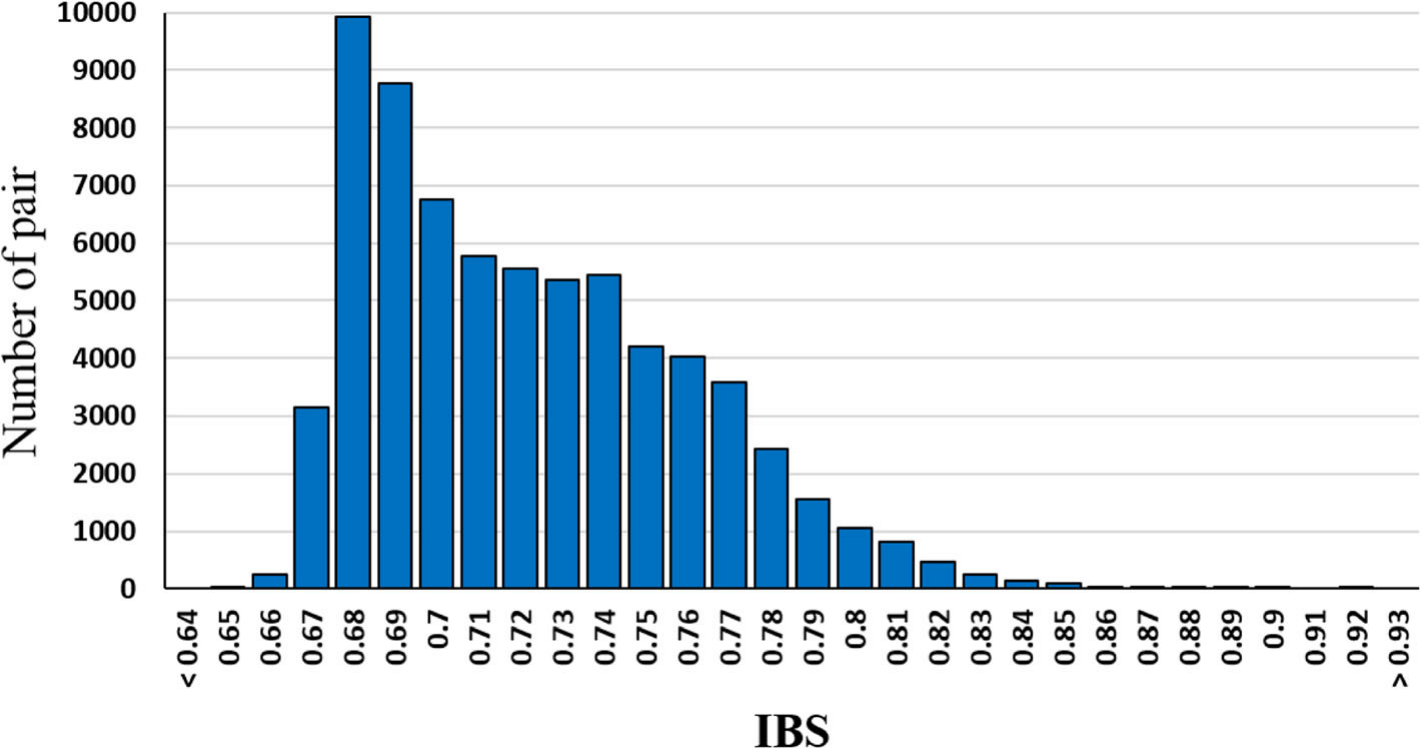
Distribution of identity by state (IBS) genetic distances amongst 374 NPGS Ethiopian accessions determined by the analysis of 27,306 unlinked (*r* ^2^ < 0.5) single nucleotides polymorphism

The preservation and effective use of the NPGS Ethiopian collection require an adequate knowledge of its genetic diversity to avoid genetic drift and phenotyping duplicate accessions. The majority of these accessions were collected more than 70 years ago and represent multiple independent germplasm collections; likewise, the seed increase process for this enormous collection (i.e., curatorial management) may contribute to problems, such as material duplication by labeling and logistic errors. We calculated the identity-by-state (IBS) genetic distance among all pairs of accessions using 27,306 unlinked SNPs (*r* ^*2*^ < 0.5) that were evenly distributed across the sorghum genome. Remarkably, of the 69,564 pairwise IBS genetic distances, only 14 exceeded 0.90 (Fig. 1), and these represented five groups of 11 closely related accessions. Indeed, no genetic profile was duplicated and the average IBS among accessions was 0.70. The homogeneity of each accession is essential for phenotyping analyses. In the GBS analysis, we detected high levels of inbreeding within accessions; the average heterozygosity (0.11) was not larger than the observed heterozygosity in previous studies of sorghum landraces [23]. Even though this study was based on 5% of the NPGS Ethiopian collection, the results indicate that its assembly and maintain has been adequate capturing and preserving its genetic diversity.

### Population structure

**Fig. 2:**
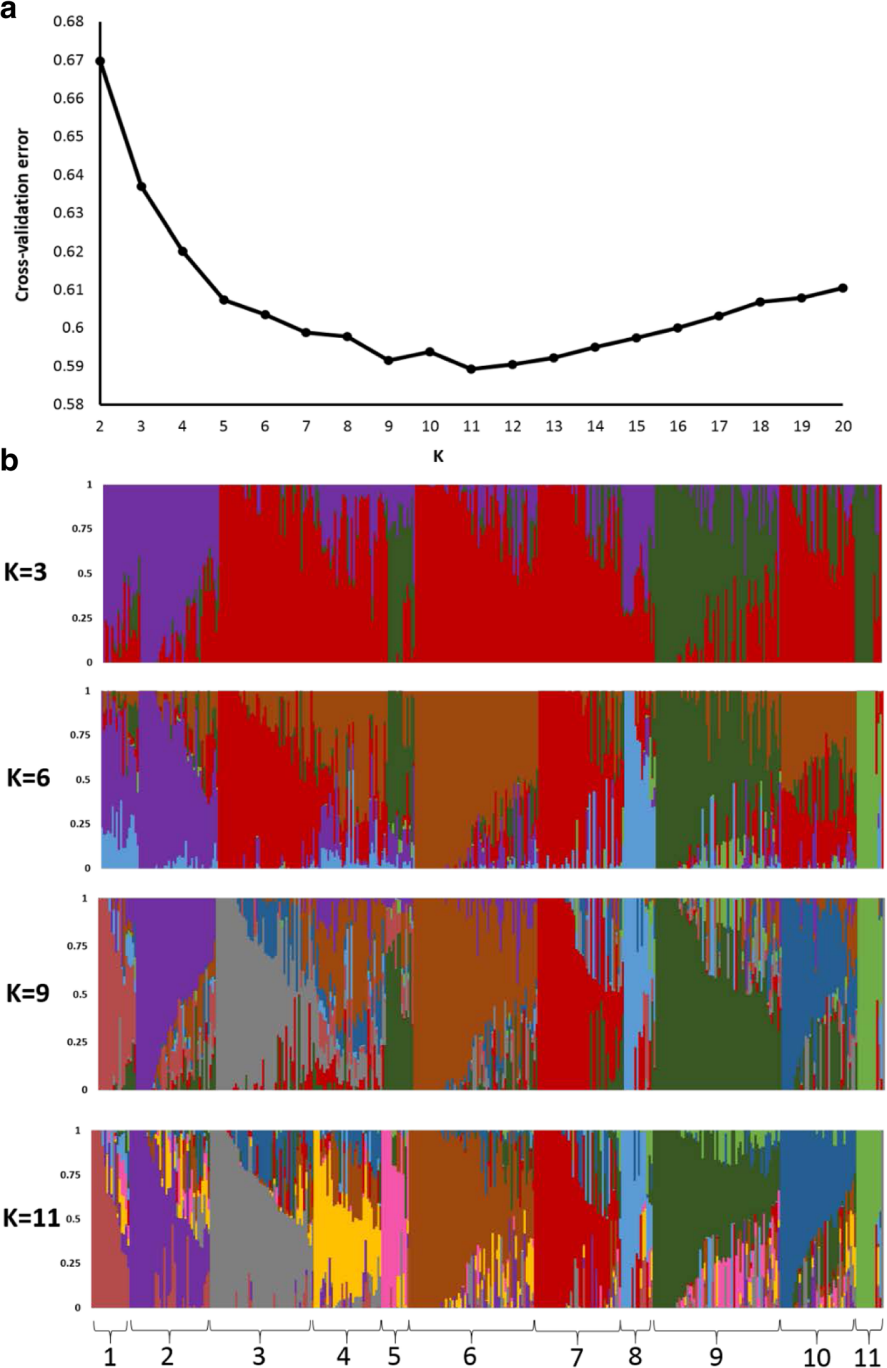
Population structure analysis of 374 NPGS Ethiopian sorghum accessions. **a** The optimal number of subpopulations was 11 based on the ADMIXTURE analysis with the cross validation for K values from 1 to 20 using 27,306 unlinked (*r* ^2^ < 0.5) SNPs distributed across the genome. **b** Hierarchical organization of genetic relatedness of the 374 Ethiopian accessions, each individual bar represents an accession

Understanding the population structure of the NPGS Ethiopian collection is imperative to establish new strategies to preserve and utilize its genetic diversity and is a prerequisite for GWAS. We inferred that there are 11 ancestral populations based on 10-fold cross-validation using ADMIXTURE [24] (Fig. 2a; referred to as populations 1 to 11). In total, we assigned 198 accessions (53%) to one of these populations with an ancestry membership coefficient of greater than 0.60, and we categorized the remaining 47% as having evidence of admixture (Fig. 2b). Indeed, the pairwise fixation index (*F*_ST_) among these populations ranged from 0.11 to 0.47, indicating a relatively high level of genetic differentiation (Table 2). We determined the effective population size (N_e_) based on the linkage disequilibrium (LD) of 58,634 SNPs with MAF > 0.05. We obtained an N_e_ of 199 individuals, which was comparable to the total number of accessions with an ancestry membership coefficient of greater than 0.60. In fact, these 198 accessions covered 90% of the observed allelic diversity (133,874 SNPs); accordingly, additional phenotyping could be limited to or initiated with these accessions. The conservation of these particular accessions is imperative to maintain the genetic diversity of this core set of the collection.

**Table 2:** Pairwise estimates of F_st_ among the 11 NPGS Ethiopian populations based on the analysis of 27,306 single nucleotide polymorphism loci among 198 accessions

	Pop 1	Pop 2	Pop 3	Pop 4	Pop 5	Pop 6	Pop 7	Pop 8	Pop 9	Pop 10
Pop 2	0.21									
Pop 3	0.30	0.33								
Pop 4	0.20	0.21	0.17							
Pop 5	0.33	0.34	0.39	0.32						
Pop 6	0.30	0.32	0.18	0.16	0.40					
Pop 7	0.33	0.36	0.11	0.20	0.44	0.22				
Pop 8	0.33	0.37	0.35	0.31	0.47	0.37	0.38			
Pop 9	0.33	0.34	0.35	0.30	0.20	0.36	0.39	0.42		
Pop 10	0.29	0.31	0.14	0.16	0.38	0.15	0.19	0.37	0.33	
Pop 11	0.44	0.44	0.45	0.44	0.39	0.46	0.50	0.55	0.26	0.47

**Fig. 3:**
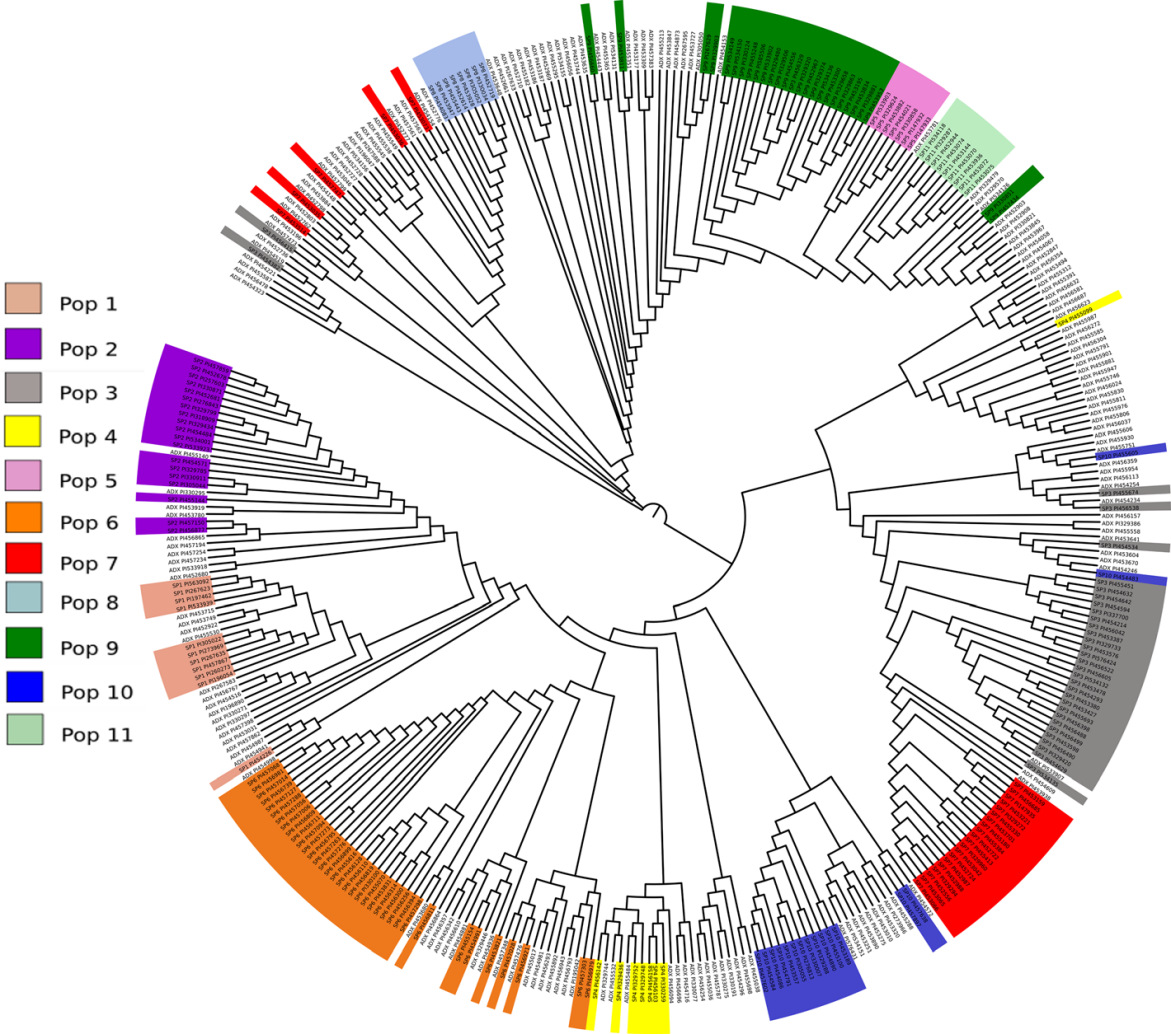
Neighbor joining tree of the 374 Ethiopian accessions where admixture accessions are not colored

We used an unrooted neighbor-joining tree to determine the genetic relationships among these populations. The results of this analysis were consistent with the previously determined population structure (Fig. 3), with 11 populations belonging to four main clades. The largest clade included populations 1, 2, 4, 6, and 10, suggesting high genetic relatedness among these populations. Additionally, populations 5, 11, and 9 formed a group. The majority of the accessions from populations 3 and 7 belonged to one common clade, while population 8 belonged to a group with three accessions from population 7. We observed accessions with evidence of admixture throughout the tree, although we detected one large cluster that included one accession from population 4. The genetic relationships among populations provide insight into the history of the observed population structure. We inferred that four founder populations may have subsequently subdivided into 11 populations. We did not detect concordance between our phylogenetic and population structure results and the 14 sorghum races present in the Ethiopian germplasm collection. Durra and the intermediate Durra-bicolor are the most frequent races, and their domestication is associated with the Ethiopian region [25]. Accessions classified as these two races were dispersed among the 11 populations, indicating that the observed population structure was most likely the result of adaptation to different Ethiopian environments. The selection of accessions for phenotype evaluations based on this population structure analysis ensures the presence of the majority of the genetic diversity present in the NPGS Ethiopian germplasm collection.

### Allelic diversity

**Fig. 4:**
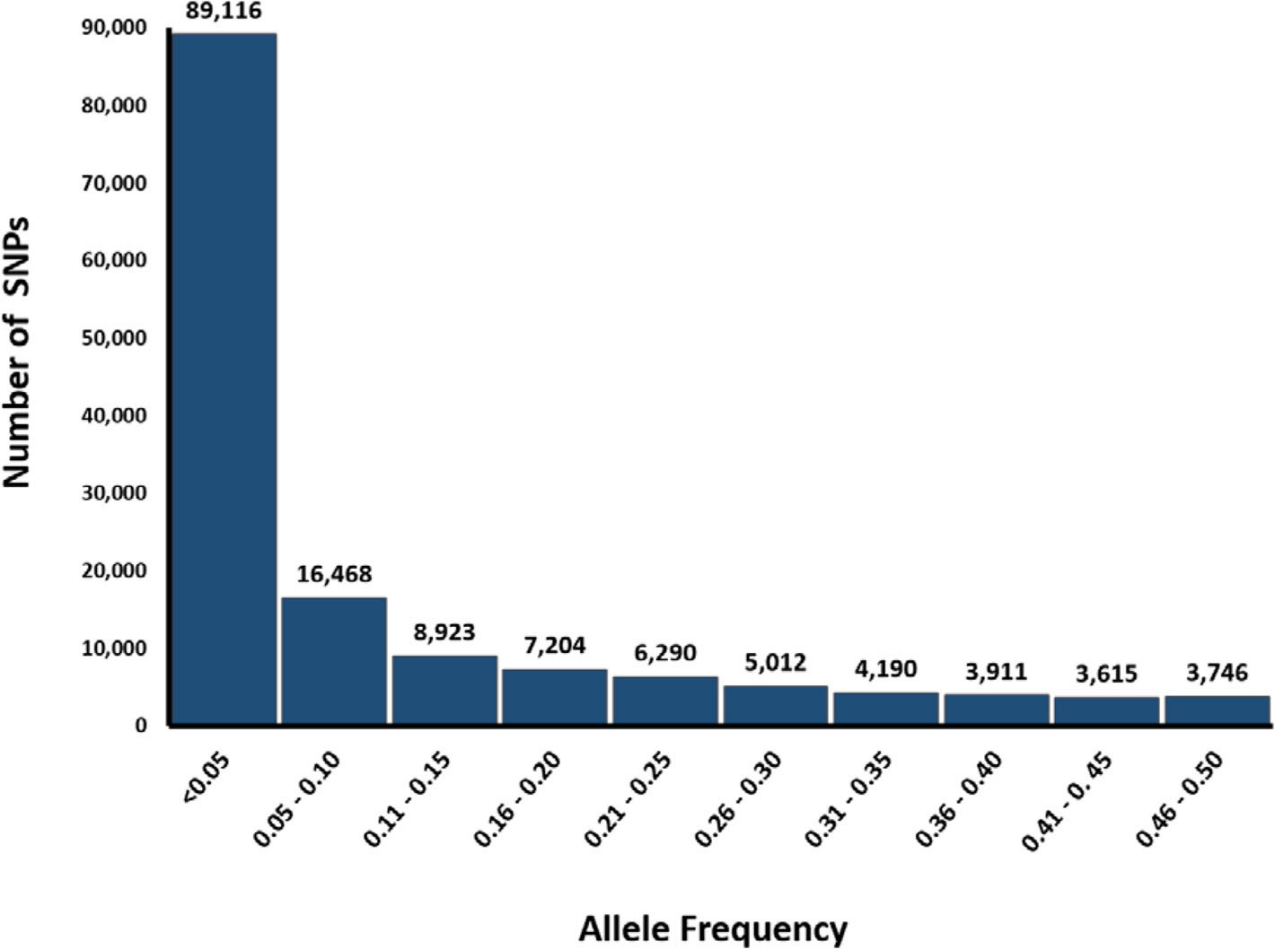
Allele frequency distribution of 148,475 single nucleotide polymorphisms among 374 NPGS Ethiopian accessions

Based on the allele frequency distribution for this NPGS Ethiopia collection core, 60% of the SNPs were rare (MAF < 0.05) (Fig. 4). A high frequency of rare alleles was also observed in a GBS analysis of 2,815 maize inbred accessions from the NPGS collection [17], confirming the high accuracy of rare allele detection using GBS. In fact, we detected all of these rare alleles in at least three accessions (MAF > 0.01). We found that the 198 representative accessions (including 90% of the SNPs) had a similar proportion of rare alleles. In the four largest populations (3, 6, 7, and 9), which included more than 25 accessions, we found that an average of 55% of alleles were rare. For populations 1, 2, and 10, which included 11 to 19 accessions, an average of 29% of alleles were rare. Since populations 4, 5, and 8 had fewer than ten accessions, we did not estimate the proportion of rare alleles. The large number of admixture accessions in the collection and the distribution of rare alleles among populations suggest these rare alleles could represent recent introgression from neighboring countries. To compare the diversity between these 11 populations, we determined the total number of private alleles in each population. We identified 10,284 private alleles that characterized these 11 populations. Populations 1 and 2 had the most private alleles (1,995 and 2,264, respectively) and these two populations (30 accessions in total) had 66% of the total allelic diversity (98,322 SNPs). Populations six and nine had 1,286 and 1,353 private alleles, respectively, and had 68% of the total allelic diversity (101,681 SNPs) with a total of 62 accessions. In the 176 accessions with evidence of admixture, we only detected 1,473 private alleles. The NPGS Ethiopian collection could be arranged into these 12 groups (11 populations and an admixture group) using a high-throughput genotyping platform based on these private alleles. The organization of this germplasm collection can improve conservation efforts and phenotypic analyses. For instance, the selection of particular populations and/or subsets of accessions that capture the majority of the allelic diversity will optimize the use of phenotyping resources. Allelic richness could be monitored as an indicator of the conservation status of its genetic diversity [26].

**Fig. 5:**
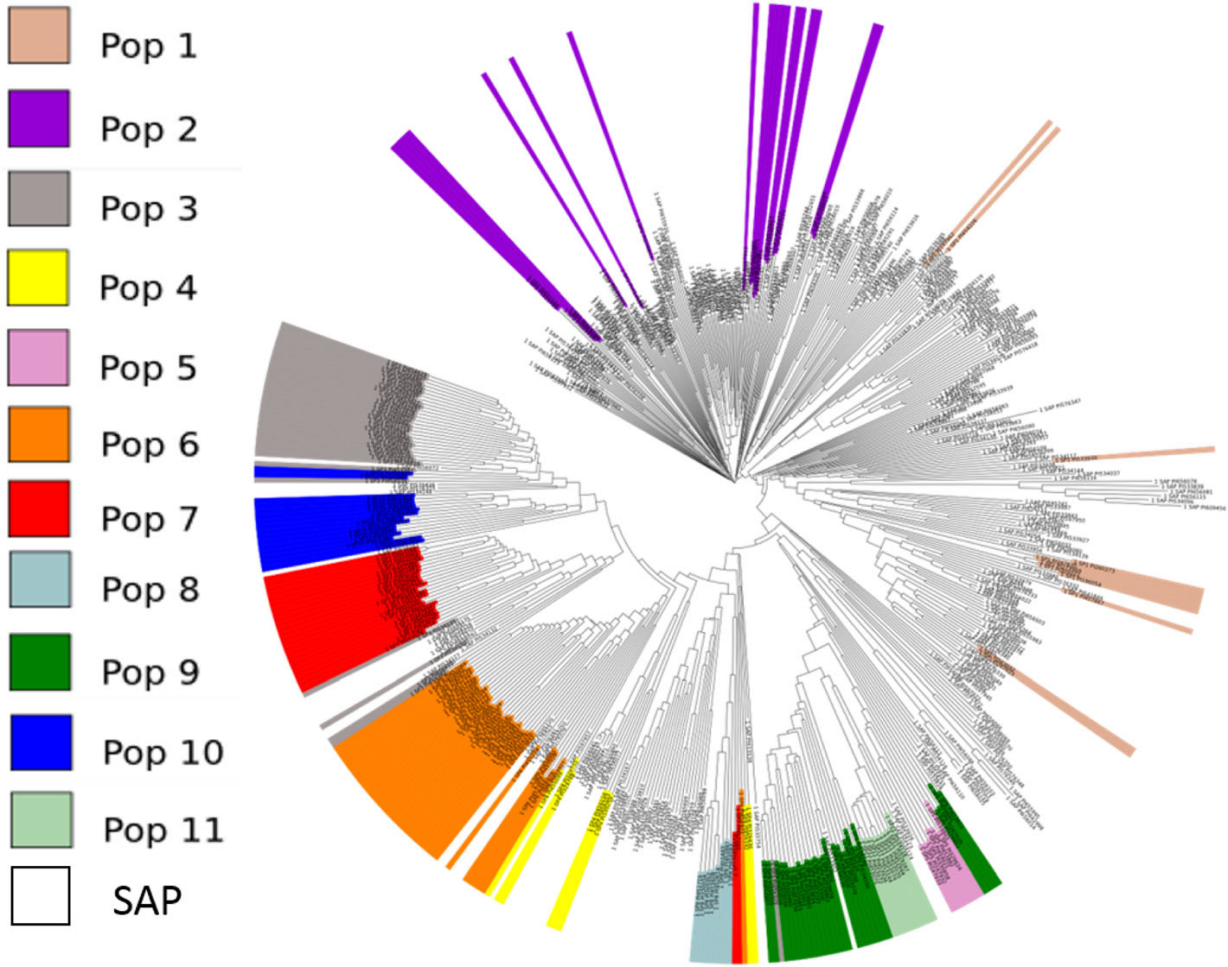
Neighbor-joining tree of the 198 representative NPGS Ethiopian accessions and 356 lines from the U.S. sorghum association panel (SAP) based on 26,026 unlinked (r^2^ < 0.5) SNPs. Colored labels represent the 11 populations present in NPGS Ethiopian germplasm

How much of the genetic diversity present in NPGS Ethiopian germplasm has been used in US breeding programs? The US SAP encompasses a wide range of sorghum germplasm that has been exploited in breeding programs and was recently genetically characterized [16]. We identified all of the common alleles between SAP and this Ethiopian core set. We detected 60,962 common alleles, of which 74% had a MAF of greater than 0.05. In fact, 20% (18,213 SNPs) of the rare alleles in the Ethiopia core set were present in SAP, while 57% (10,382) had a MAF of greater than 0.05. In total, 59% of the 10,284 private alleles that characterize the 11 Ethiopian populations were present in SAP. However, only the private alleles from populations 1 and 2 were adequately represented in SAP (87 and 80%, respectively). In fact, the neighbor-joining cluster analysis of SAP and the 11 Ethiopian populations based on 26,026 unlinked SNPs showed that populations 1 and 2 are completely integrated in SAP (Fig. 5), suggesting these two populations have been used extensively in breeding programs. The other nine Ethiopian populations expanded the genetic diversity of SAP accessions originally from Ethiopia and India. Hence, the NPGS Ethiopian collection is a germplasm resource that could be used to support and increase the genetic diversity in SAP. The integration of particular Ethiopian accessions from populations 3 to 11 into SAP will be useful for the discovery of new agronomically important alleles and to advance the use of this germplasm collection.

### Genetic diversity and signatures of selection

The management of germplasm collections is intended to maintain the maximum possible genetic diversity of the crop. We measured the levels of genetic diversity and divergence among populations in the NPGS Ethiopia collection based on LD (as estimated by *r* ^*2*^) and *F*_ST_ across the genome. In the Ethiopia collection, we observed an average *r* ^*2*^ of ≤0.2 at distances of greater than 6 kb (Additional file 1: Figure S1). This decay in LD was more rapid than the decay observed in previous analyses of 330 landraces from different African countries and the SAP, in which LD extended from 20 to 50 kb [16]. The rapid decay in LD among Ethiopian accessions could be explained by the early establishment of these populations after the domestication of sorghum. Moreover, many accessions had evidence of admixture, which reduces the average length of LD blocks, reflecting a mixture of ancient and new Ethiopian landraces. Certainly, the high number of recombination events is associated with the phenotype diversity of this germplasm, and can be useful for the genomic dissection of agronomically important traits by GWAS; however, deep sequencing coverage may be required to cover all genomic blocks.

**Fig. 6:**
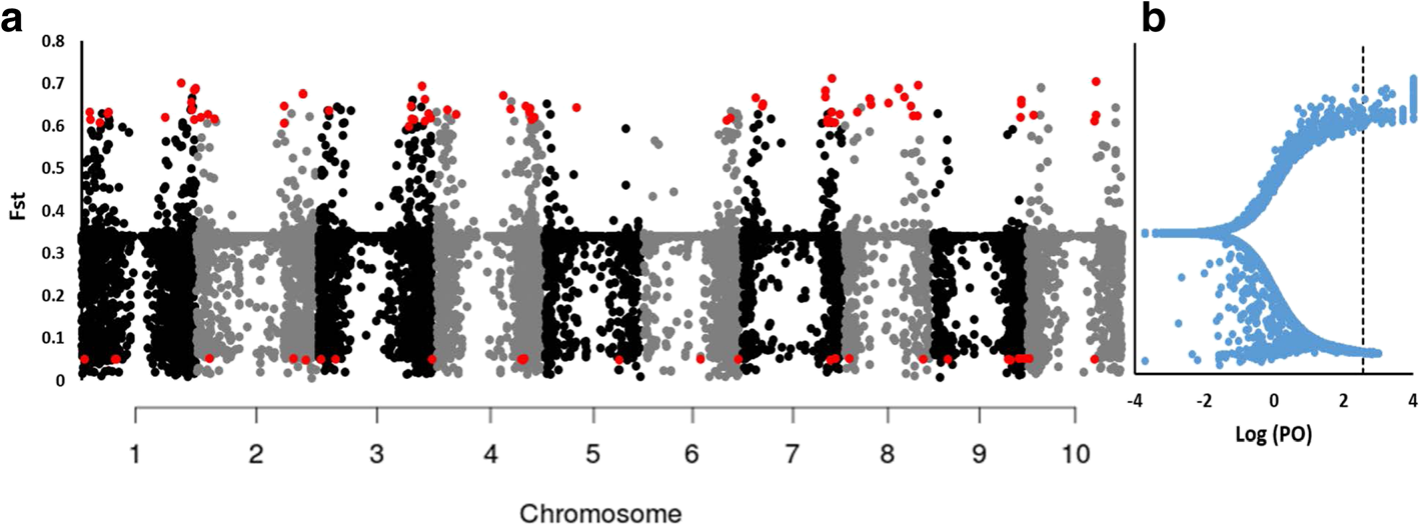
BAYESCAN result for the analysis of 27,306 unlinked SNPs distributed among 11 Ethiopian sorghum populations for outlier prediction. **a** The distribution of SNPs across the sorghum genome. Red dots represents Fst outliers. **b** BayeScan analysis for outlier prediction with a false discovery rate (FDR) of *p* < 0.001 (*broken line*)

The population structure of a plant is determined by abiotic and biotic factors that influence selection [27]. The high genetic differentiation index (*F*_ST_) between these 11 Ethiopian populations (Table 2) indicates selection in particular environmental conditions. The identification of traits that define each of these populations is imperative to improve the use of this germplasm; agronomic traits that are at high frequencies within a particular population can be screened. In this regard, the genetics underlying the observed population structure may reveal genomic regions that are involved in the selection of particular traits. To identify genomic regions that may be under selection pressure, we used the *F*_ST_ outlier method implemented in BayeScan v.2.1 [28]. This approach distinguishes between loci that diverged via random drift and those that diverged via selection. We detected 99 SNPs that diverged by selection, of which 72 SNPs were consistent with diversifying selection and 27 SNPs were consistent with balancing selection (Fig. 6). We observed these signatures of selection across the whole genome, but particularly in sub-telomeric gene-rich regions. We detected diversifying selection on *Tannin1* [29] (*F*_ST_ = 0.62). Tannins have diverse biological functions, including protection against pathogenic bacteria and fungi and predation by herbivorous animals [30]. We detected additional *F*_ST_ outlier SNPs with a broad range of gene ontology annotations, including metabolic process (glycosyl transferase), signal transduction (auxin response factor), DNA damage repair, and retrotransposon proteins. Natural selection contributes more substantially to the structure of Ethiopian sorghum populations than selection by farmers.

### Phenotypic diversity

The NPGS Ethiopian germplasm collection is the largest group of accessions in the sorghum collection; it includes 11 populations based on a genetic analysis. The phenotypic characterization of these populations is necessary to deeply understand the population structure and improve the use of the collection. We characterized these populations with respect to agronomic traits that might be associated with ecogeographical variation and the agricultural system of Ethiopia as well as other traits of potential interest to the sorghum breeding community.

The phenotypic diversity of Ethiopian germplasm was associated with the observed population structure. In fact, among the 11 Ethiopian populations, we observed differences in 14 evaluated traits, with an average of six groups per trait (Tables 3 and 4). These results confirmed the observed diversifying selection in the genetic analysis since contrasting phenotypes characterize these populations (i.e., high, low, and intermediate trait values). In fact, each population had a unique phenotypic profile, suggesting that they represent different environmental conditions affecting selection. For instance, in population 5, we observed evidence of selection for high biomass and small seed size, and we observed higher levels of tannins contentin population 9. Certainly, the population structure could reflect agro-environmental regions in Ethiopia and/or agronomically important traits, and could be used to establish better strategies to select accessions for phenotype screening.

**Table 3:** Phenotypic traits for the 11 NPGS Ethiopian subpopulations

			Panicle					
References	Flowering^1^	Plant height^2^	Length^3^	Diameter^4^	Ratio^5^	Seed Size^6^	Dhurrin^7^	G15^8^
RTx 2911	72 ± 17	105 ± 32	17.3 ± 5.8	2.9 ± 1.4	6.8 ± 3.1	4.0 ± 0.7	21.2 ± 8.0	*n.a*
SC719	64 ± 08	111 ± 19	18.4 ± 4.4	2.7 ± 1.2	7.7 ± 2.8	5.2 ± 1.6	14.4 ± 0.9	*n.a*
PI 610727	*n.a*	*n.a*	*n.a*	*n.a*	*n.a*	*n.a*	*n.a*	53.0 ± 6.0
RTx436	*n.a*	*n.a*	*n.a*	*n.a*	*n.a*	*n.a*	*n.a*	00.0 ± 0.0
*Ethiopia germplasm*								
Population	***	***	***	***	***	***	***	*n.s.*
Location	***	***	**	*n.s.*	*n.s.*	**	*n.a.*	*n.a.*
Pop. x Loc.	*n.s.*	*n.s.*	*n.s*	*n.s.*	*n.s.*	*n.s.*	*n.a.*	*n.a.*
R^2^	0.64	0.51	0.45	0.23	0.40	0.40	0.21	0.11
C.V.	10.01	22.13	29.23	41.41	37.42	22.08	82.51	92.01
*Fisher LSD test*								
Population 1	73 ± 09 d	138 ± 25 e	19.6 ± 2.4 cde	4.2 ± 1.0 cd	5.3 ± 1.7 bc	3.8 ± 1.1 bc	19.8 ± 30.8 bcd	18.2 ± 35.9
Population 2	65 ± 07 e	159 ± 35 de	18.2 ± 4.8 def	4.4 ± 1.5 cd	4.2 ± 1.6 cd	4.4 ± 0.6 ab	37.1 ± 23.0 a	40.9 ± 30.0
Population 3	79 ± 04 b	203 ± 28 b	20.9 ± 6.1 cd	4.7 ± 1.1 cd	4.6 ± 1.3 bc	4.6 ± 0.9 ab	11.4 ± 18.3 d	27.7 ± 39.5
Population 4	65 ± 06 e	145 ± 17 de	11.3 ± 2.7 g	4.1 ± 1.2 d	3.0 ± 0.8 def	4.2 ± 0.7 ab	17.3 ± 05.1 bcd	50.6 ± 37.3
Population 5	77 ± 01 cd	265 ± 37 a	35.4 ± 5.9 a	7.5 ± 2.0 a	5.0 ± 1.1 bc	2.8 ± 0.2 d	14.9 ± 10.1 cd	36.6 ± 25.7
Population 6	74 ± 06 cd	172 ± 29 cd	23.4 ± 4.5 cd	4.4 ± 1.7 cd	6.0 ± 1.9 ab	4.6 ± 0.9 ab	14.5 ± 12.7 d	33.0 ± 32.3
Population 7	78 ± 08 cd	192 ± 35 bc	15.5 ± 6.5 efg	5.5 ± 1.9 bcd	2.9 ± 0.5 ef	4.5 ± 0.6 ab	28.7 ± 21.5 abc	12.4 ± 16.5
Population 8	85 ± 08 a	199 ± 36 bc	13.4 ± 1.9 fg	6.5 ± 2.0 ab	1.9 ± 0.3 f	3.9 ± 0.6 abc	30.7 ± 09.4 ab	36.3 ± 22.4
Population 9	75 ± 04 cd	192 ± 25 bc	24.2 ± 4.7 bc	6.9 ± 2.7 ab	4.2 ± 1.3 cde	2.9 ± 0.4 d	18.2 ± 09.9 bcd	42.9 ± 21.4
Population 10	76 ± 07 cd	203 ± 26 b	29.3 ± 7.6 b	4.5 ± 1.3 cd	6.9 ± 1.6 a	4.6 ± 0.7 a	14.7 ± 08.1 d	24.6 ± 27.1
Population 11	84 ± 04 ab	200 ± 19 bc	15.5 ± 3.4 efg	5.9 ± 1.4 abc	2.7 ± 0.2 f	3.3 ± 0.3 cd	13.8 ± 06.7 d	21.1 ± 15.0

Phenotype evaluations were completed at Isabela and Mayaguez, Puerto Rico in 2013 and 2014, respectively. Means followed by a common letter are not significantly different according to Fisher’s least significant different test

^1^Flowering time refers to days to 50% flowering of the plot

^2^Plant height (cm) refer to the distance from the base of the main stalk to the top of the panicle without sorghum converted accessions

^3^Panicle length (cm) refers to the distance from the base to the top of the panicle

^4^Panicle diameter (cm) refers to the wider region of the panicle

^5^Ratio refers to the length/diameter

^6^Seed size refers to the volume of 100 seeds

^7^Dhurrin (μg/cm^2^) content in leaf measure at post-flowering stage using HPLC [53]

^8^G15 refers to the percent of 30 seeds that germinated after 72 h at 15 °C in growth chamber

n.s., ** and *** refers to no significant, and significant effects at *p* ≤ 0.01 and .005, respectively

C.V. refer to coefficient of variation

**Table 4:** Seed composition traits for the 11 NPGS Ethiopian subpopulations

References	Protein^1^	Fat^1^	Fiber^1^	Ash^1^	Starch^1^	Phenols^2^	Tannins^3^	3-DOAs^4^
RTx 2911	11.4 ± 1.0	1.6 ± 0.2	1.4 ± 0.1	1.3 ± 0.00	66.6 ± 1.2	05.1 ± 1.7	00.0 ± 00.0	36.8 ± 07.8
SC719	10.0 ± 1.3	2.9 ± 0.5	1.9 ± 0.2	1.3 ± 0.10	66.4 ± 1.0	06.3 ± 1.7	11.4 ± 02.8	22.0 ± 03.9
*Ethiopia germplasm*								
Population	***	**	***	***	n.s.	***	***	***
R^2^	0.35	0.22	0.35	0.54	0.11	0.52	0.46	0.34
C.V.	16.77	18.50	11.29	6.15	2.56	66.00	94.57	64.80
*Fisher LSD test*								
Population 1	10.7 ± 1.6 c	2.7 ± 0.6 c	1.6 ± 0.1 bc	1.25 ± 0.04 abc	66.0 ± 1.4	09.6 ± 5.3 b	16.9 ± 14.1 b	35.1 ± 21.9 cd
Population 2	13.0 ± 3.5 bc	2.8 ± 0.9 bc	1.6 ± 0.2 abc	1.33 ± 0.08 a	64.9 ± 2.5	08.4 ± 4.0 bc	13.5 ± 09.0 bc	29.5 ± 22.0 cd
Population 3	13.1 ± 2.1 bc	2.8 ± 0.3 bc	1.6 ± 0.2 abc	1.27 ± 0.07 abc	65.1 ± 1.5	05.2 ± 5.8 bcd	07.4 ± 11.8 bcd	39.1 ± 29.3 bcd
Population 4	13.4 ± 2.9 b	2.8 ± 0.4 c	1.6 ± 0.0 bc	1.23 ± 0.12 bc	62.9 ± 2.0	06.2 ± 2.4 bcd	02.3 ± 03.3 cd	46.7 ± 14.3 bcd
Population 5	10.8 ± 0.6 c	3.3 ± 0.9 abc	1.5 ± 0.1 cd	1.31 ± 0.08 ab	65.5 ± 0.6	10.0 ± 1.7 b	18.9 ± 05.1 b	15.4 ± 22.6 d
Population 6	12.8 ± 2.2 bc	2.7 ± 0.5 c	1.7 ± 0.1 ab	1.31 ± 0.05 ab	64.8 ± 1.5	05.1 ± 3.3 bcd	07.3 ± 06.8 bcd	31.9 ± 14.4 cd
Population 7	14.0 ± 2.3 b	3.1 ± 0.5 abc	1.5 ± 0.2 bc	1.29 ± 0.06 ab	64.0 ± 1.7	03.2 ± 3.8 cd	03.1 ± 06.7 cd	54.1 ± 32.5 abc
Population 8	15.1 ± 1.3 ab	3.4 ± 0.2 ab	1.6 ± 0.1 bc	1.20 ± 0.11 c	64.0 ± 1.0	09.5 ± 9.7 b	15.9 ± 20.3 b	69.1 ± 18.3 ab
Population 9	16.9 ± 2.1 a	3.5 ± 0.5 a	1.3 ± 0.2 d	1.07 ± 0.13 d	64.3 ± 1.4	18.1 ± 9.7 a	33.5 ± 15.5 a	82.1 ± 44.0 a
Population 10	13.8 ± 2.9 b	3.0 ± 0.9 abc	1.6 ± 0.2 abc	1.29 ± 0.06 ab	64.3 ± 2.2	06.2 ± 6.7 bcd	10.0 ± 13.8 bcd	23.2 ± 21.9 cd
Population 11	13.1 ± 0.8 bc	3.5 ± 0.2 a	1.8 ± 0.1 a	1.30 ± 0.02 ab	64.8 ± 0.8	00.9 ± 0.8 d	00.0 ± 00.1 d	29.1 ± 17.2 cd

Seed composition traits were determined based on near-infrared (NIR) spectroscopy calibration curve (Dykes et al. [45]) from fresh seed obtained from Isabela, Puerto Rico in 2013. Means followed by a common letter are not significantly different according to Fisher’s least significant different test

^1^Values are based on percent

^2^Total phenols based on milligram of gallic acid equivalent (GAE)/gram

^3^Condensed tannins based on milligram of catechin equivalents (CE)/gram

^4^3-Deoxyanthocyanidins content based on absorbance (abs)/milliliter/gram

n.s., ** and *** refers to no significant, and significant effects at p ≤ 0.01 and .005, respectively

C.V. refer to coefficient of variation

To improve our understanding of the selection process underlying the observed population structure, we used a principal component analysis (PCA) to identify the relative importance of these traits in shaping phenotype diversity. The first four principal components accounted for approximately 60.5% of the total phenotype diversity, and seed compositional traits had the largest loading coefficients (Table 5). In fact, phenol, protein, tannin, and 3-DOA contents had highly similar degrees and directions for PC1 loading. The high diversity of these four seed compositional traits may be explained by opposing selection pressures driven by humans and natural conditions. For instance, high levels of tannins and phenols are associated with grain mold resistance, but these two compounds are unacceptable in seeds for human consumption [31]. Agronomical traits, such as plant height (PH), panicle length and diameter (PL and PD), and flowering, showed positive loading values for PC2, while the dhurrin content (associated with drought tolerance) had loading effects in PC3. These results indicate that seed compositional traits define Ethiopian sorghum cultivars, while agronomical traits have a secondary role owing to the undeveloped agricultural system in the country. Hence, the germplasm includes valuable alleles for seed compositional traits for sorghum breeding programs. In fact, the high genetic and phenotypic diversity within the Ethiopian germplasm is the result of a balance between both artificial and natural selection in each population.

**Table 5:** Principal component analysis of 16 phenotype traits evaluated in 374 accessions from NPGS Ethiopian germplasm collection

Trait	PC1	PC2	PC3	PC4
Phenols^1^	0.37*	−0.08	−0.31	0.09
Protein^2^	0.35*	−0.05	0.30*	0.25
Tannins^3^	0.36*	−0.05	−0.33	0.08
3-DOAs^4^	0.31*	−0.21	0.20	0.03
Plant height^5^	0.08	0.57*	0.12	−0.10
Length^6^	0.09	0.57*	−0.21	0.28
Diameter^7^	0.29	0.34*	−0.02	−0.48
Dhurrin ^8^	0.10	−0.03	0.38*	0.04
Ratio^9^	−0.13	0.18	−0.18	0.70*
Flowering^10^	0.05	0.28*	0.23	−0.10
Seed Size^11^	−0.31	0.00	0.20	0.07
Fat^2^	0.25	0.15	0.18	0.04
Fiber^2^	−0.29	0.13	−0.05	−0.06
Ash^2^	−0.33	0.13	0.26	0.03
Starch^2^	−0.20	0.03	−0.47	−0.29
G15	0.01	−0.14	−0.08	−0.02
% Variance	24.4	13.6	12.6	9.90
% Cumulative	24.4	38.0	50.6	60.5

Phenotype evaluations were completed at Isabela and Mayaguez, Puerto Rico in 2013 and 2014

^1^Total phenols based on milligram of gallic acid equivalent (GAE)/gram

^2^Values are based on percent

^3^Condensed tannins based on milligram of catechin equivalents (CE)/gram

^4^3-Deoxyanthocyanidins content based on absorbance (abs)/milliliter/gram

^5^Plant height refer to the distance from the base of the main stalk to the top of the panicle without sorghum converted accessions

^6^Panicle length refers to the distance from the base to the top of the panicle

^7^Panicle diameter refers to the wider region

^8^Dhurrin content in leaf measure at post-flowering stage using HPLC [53]

^9^Ratio refers to the length/diameter

^10^Flowering time refers to days to 50% flowering of the plot

^11^Seed size refers to the volume of 100 seeds

* most important variable in the component

### GWAS studies

The Ethiopian NPGS germplasm collection is a highly genetically and phenotypically diverse public germplasm with important applications for the improvement of sorghum breeding programs. Despite the large size of this collection, the use of molecular markers associated with important agronomic traits could facilitate the identification of valuable accessions. Therefore, we explored the potential use of these low-coverage data and the population structure analysis for GWAS. We analyzed seed tannin content and PH, two extensively studied traits [16, 29]. The SNP with the strongest association with tannin content (S4_61667908; *p* < 8.2^−10^; Additional file 1: Figure S2a) was the same as the one found in an analysis using the SAP [16] and explained 13.7% of the genetic variance. This SNP was not in complete LD with the *tan1* null allele [29], as observed in the SAP [16]. Additionally, 22 converted dwarf tropical lines in the panel were used to detect dwarfing genes on chromosomes 9 (*dw1*), 7 (*dw3*), and 6 (*dw2*) (Additional file 1: Figure S3); these loci were introgressed during the conversion of these lines [32] and explained 27% of the genetic variance. Based on Q–Q plots, both traits demonstrated associations when controlling for population structure and genetic relatedness using a compressed mixed linear model, decreasing the identification of spurious associations (Additional file 1: Figure S4).

**Fig. 7:**
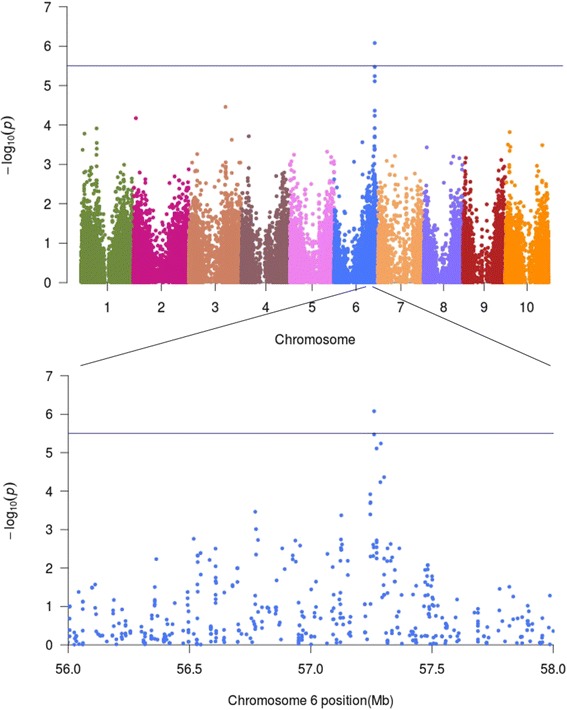
GWAS for plant height using a subset of 352 Ethiopian accessions from NPGS germplasm collection

To identify novel loci in exotic germplasm, we analyzed PH and flowering time (FL) without including the 22 converted dwarf tropical lines. We detected a significant association between PH and the distal region of chromosome 6 (S6_57260888; *p* < 8.3^−7^; Fig. 7). This loci explained 6.8% of the genetic variance while the genetic relatedness among accessions (i.e., pseudo-heritability) accounted for 34.5% of the genetic variance indicating that a large portion of the variation is still uncharacterized. Although we did not identify a candidate gene in the region or overlap with a previously detected QTL, the region deserves further study. We did not detect a significant association for FL using the whole subset or the subset lacking the converted lines. In an analysis of other agronomical traits (e.g., PL, width and ratio, seed size, and cold tolerance), we did not identify significant associations. Indeed, in GWAS for some of these traits using the SAP, significant associations have not been detected [33, 34]. The low heritability and phenotype variation for some of these traits, limited number of accessions, low-coverage data, and complexity of the traits could affect the detection of significant associations.

**Fig. 8:**
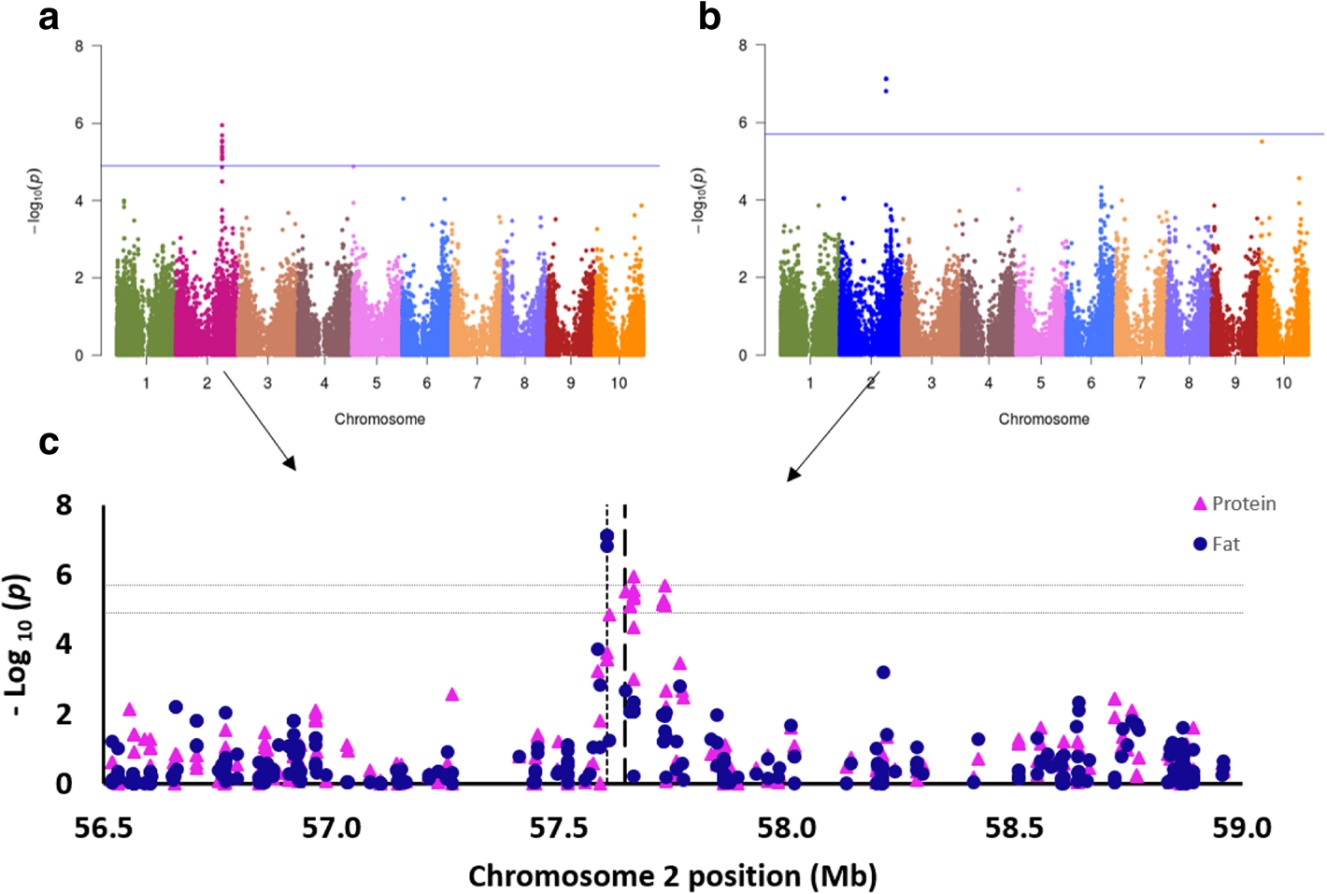
GWAS for seed protein and fat content using a subset of 374 Ethiopian sorghum accessions from the NPGS germplasm collection. **a** GWAS for protein content, **b** GWAS for fat content, **c** Chromosome 2 region associated with fat and protein

Based on a GWAS of seed compositional traits, we identified novel loci for protein and fat content, and confirmed a previous result for total phenol content. We obtained similar results for total phenol and tannin contents; SNP S4_61667908 demonstrated the most significant association for these traits (*p* < 1.4^−10^; Additional file 1: Figure S2b). A significant correlation between these two traits was also observed in the SAP, in which total phenol content was associated with *Tannin1* [35]. A genomic region in chromosome 2 was associated with fat and protein content (Fig. 8a and b). Even though we detected a significant correlation between the fat and protein contents (*r* ^*2*^ = 0.54; *p* < 0.0001), we identified two loci in the GWAS that were 57 kbp apart (Fig. 8c). In fact, we detected the strongest associations for fat and protein content with the SNPs S2_57605132 (*p* < 7.6^−8^) and S2_57662361 (*p* < 1.2^−6^), respectively, which explained 11.5 and 9.6% of the genetic variance, respectively. Since the genetic relatedness among accessions only accounted for 23.3% (fat) and 21.1% (protein) of the genetic variance other important loci are still unidentified. These SNPs were located within the genes Sb02g023740.1 (unknown protein) and Sb02g023770.1 (cytochrome P450), which have not been associated with particular phenotypes in sorghum or *Arabidopsis*. Additional phenotype and genotype analyses are required to verify these associations. This Ethiopian core set and its genetic characterization could provide a basis for the discovery of important loci in this exotic germplasm.

## Discussion

At present, only a minor portion of the genetic diversity in the NPGS Ethiopian collection is used in sorghum breeding programs. The large size of this particular collection and the lack of genetic and phenotypic data have contributed to the limited use of this germplasm. Therefore, the genomic characterization presented herein should encourage and facilitate its use by breeders and geneticists worldwide. The combination of this genomic information with large-scale phenotyping efforts could be used to mine novel alleles for the improvement of agronomic traits. The development of new cultivars with novel alleles from Ethiopian collections is a promising strategy to translate this genetic diversity into agriculture benefits.

Untapping the genetic variation in the NPGS Ethiopian germplasm for sorghum improvement is challenging and requires precise strategies. Historically, the integration of tropical exotic germplasm into sorghum breeding programs has relied on the use of adapted exotic germplasm (i.e., converted germplasm) for temperate grain production regions via the introgression of dwarfing and early maturity genes [36]. Today, more than 1,000 exotic sorghum lines are adapted to temperate regions, of which approximately 20% originated in Ethiopia [32]. Nevertheless, the rapid fixation of temperate-adapted alleles in early backcross generations reduces genetic variation in multiple genomic regions; in fact, the genetic variation in chromosome 6 is minimal within converted sorghum germplasm [32]. Therefore, breeding schemes that increase recombination in early generations could be used to develop temperate-adapted germplasm. In this regard, a multi-parent advanced generation intercross design [37] or the employment of various cycles of recurrent genotypic selection should be adequate to exploit the genetic diversity of Ethiopian germplasm. For instance, an initial phenotypic evaluation (i.e., for a trait of interest) may be completed to identify superior and genetically diverse Ethiopian accessions in the core set and these accessions may be crossed with a temperate-adapted germplasm. The progeny can be systematically intercrossed and/or selfed in tropical regions during early generations and genotyped to maintain temperate-adapted alleles (e.g., related to dwarfing and maturity). Superior families can be evaluated in temperate regions for traits of interest and to fix the temperate-adapted alleles. Indeed, previous phenotypic analyses of these types of populations in *Arabidopsis* [38], rice [39], and wheat [40] identified transgressive segregation, which is valuable for breeding.

Today, the SAP is widely used for the genomic dissection of multiple agronomically important traits [16, 33, 34, 41]. This diversity panel, which includes worldwide sorghum accessions, captures a large portion of sorghum genetic diversity. However, the limited number of Ethiopian accessions (31) does not adequately represent the genetic diversity in the NPGS Ethiopia germplasm collection (Fig. 5). Moreover, these accessions have lower genetic diversity than the original exotic germplasm, since they are temperate-adapted lines developed by the introgression of common genomic regions associated with photoperiod sensitivity and dwarfing [32]. The expanded use of the NPGS collection requires a size reduction to expedite the screening process. Our genomic characterization clearly indicated that the NPGS Ethiopian germplasm collection could be reduced by 50% while maintaining more than 90% of the total genetic diversity. Certainly, a continuous reduction in GBS data makes it possible to generate SNP data on the whole collection at lower coverage (1×) to establish a precise core set.

Germplasm conservation is imperative for continual agricultural improvements. Studies of the genetic relatedness among all ex situ accessions in germplasm collections and genetic redundancy should improve conservation efforts and practical applications. GBS is rapidly becoming the standard choice for genetic studies and germplasm characterization. Hence, in the near future, multiple independent GBS analyses of sorghum accessions from different germplasm collections will likely be integrated into a meta-analysis to identify duplicates and closely related accessions among worldwide germplasm collections. In this regard, a multi-institutional effort is necessary to systematically explore the genetic diversity of all ex situ sorghum collections to broaden the genetic base of breeding programs worldwide. Indeed, the preservation of redundant germplasm in ex situ collections is a waste of limited resources [42] and does not promote the goal of improving the germplasm.

## Conclusion

The NPGS Ethiopian sorghum germplasm is a highly genetically and phenotypically diverse germplasm collection comprising 11 populations. The genetic diversity of the germplasm is largely unused in sorghum breeding programs. Our results provide sorghum breeders and geneticists worldwide with knowledge and tools to utilize and conserve this germplasm. In fact, these results will facilitate resource optimization by improving experimental designs based on population structure and genetic diversity. Moreover, this genomic characterization is the first step toward the development of a NPGS sorghum core collection based on genetic profiles.

## Methods

### Germplasm

The 7,237 Ethiopian sorghum accessions in the current NPGS collection were grouped according to the GRIN database into the 16 known sorghum races and one unknown category (Table 1). Sorghum race classification was selected as the classifiable variable since the information is available for 72% of the accessions [19]. Subsequently, a core set of 374 accessions was established using a stratified sampling method based on these 17 categories (i.e., random selection within each group/category). The final core set included 352 exotic germplasm accessions and 22 converted tropical lines that are photoperiod-insensitive produced by crossing exotic lines and modern US cultivars. In addition, the lines RTx2911 and SC719 were included in the phenotypic analysis as references [43].

### Phenotypic analysis

The 374 accessions and two references were evaluated at Isabela and Mayaguez, Puerto Rico during the short day-length season for 2 consecutive years (November 2013 to March 2014 and October 2014 to February 2015). In both locations, a completely randomized design with plots of 1.8 m in length with 0.9 m between rows was used. Plants were maintained using standard management practices, and weeds were controlled with mechanical tillage and hand hoeing.

FL, PH, PL and PD, the ratio of PL/PD, and seed size were obtained for each accession/plot at both locations. FL was defined as the number of days until 50% of the plants within a plot reached anthesis. PH referred to the average distance from the base of the main stalk (i.e., soil) to the top of the panicle at maturity for two representative plants per accession/plot. PL and PD referred to the distance from the first rachis to the top of the panicle, and to the widest section of the panicle [44], while RA was defined as PL/PD. Seed size was measured based on the volume of 100 seeds. Indeed, the volume of 100 seeds was significantly correlated with seed length (*r* ^*2*^ = 0.81), width (*r* ^*2*^ = 0.92), diameter (*r* ^*2*^ = 0.78), and area (length × width × diameter; *r* ^*2*^ = 0.95) (*unpublished observations*). Thus, seed size referred to the average volume of 100 seed from three representative panicles per accession.

A compositional analysis of whole seeds was performed using near-infrared (NIR) spectroscopy [45] in 2013. Briefly, 25–50 g of fresh seeds from each accession was scanned using a FOSS XDS NIR (FOSS North America, Eden Praire, MN, USA) spectrometer with a wavelength of range of 400–2,500 nm, and collection spectra data analyzed using ISIscan software (Version 3.10.05933). Based on near-infrared calibration curves, the percent protein, fat, fiber, ash, total phenols (mg gallic acid equivalent/g), condensed tannins (mg catechin equivalents/g), and 3-DOA (absorbance/ml/g) were estimated for each accession.

Early-season cold tolerance was determined using the average cold germination (G72) for fresh seeds from 2013 according to the procedures recommended by the Association of Official Seed Analysts [46]. The lines PI610727 and RTx436 were included in the early-season cold tolerance evaluation as resistant and susceptible references, respectively [47]. Briefly, average G72 values were recorded by sowing ten seeds at 12 °C in polystyrene Petri dishes lined with filter paper moistened with distilled water. The number of germinated seeds after 72 h was determined visually based on the presence or absence of an emerged radicle. Each accession was replicated three times using a completely randomized design. Seeds were allowed to germinate and grow at 12 °C under full light conditions for 3 weeks.

Leaf dhurrin content is a quantitative measure of the level of pre- and post-flowering drought tolerance [48]. In 2013, five representative plants from each accession were punched at anthesis midway between the blade tip and base from the newest fully developed leaf, excluding the flag leaf. The dhurrin content in the leaf samples was extracted and quantified by high-performance liquid chromatography according to Burke et al. [48].

The combined data over all locations were used to standardize the 16 traits to a mean of zero and a standard deviation of one for multivariate analysis. PCA was performed using the standardized data with a covariance matrix in SAS. Likewise, analysis of variance (ANOVA) were performed using the subset of 198 accessions assigned to the 11 subpopulations. The linear effect model for such ANOVA was the following: *Y* = μ + *L* + *P* + *L* x *P* + *e*; where *Y* is the trait, μ is the common effect, *L* is the location effect, *P* is the population effect, *L* x *P* is the year × location effects and *e* is the plot to plot variation within populations. The ANOVA for traits with one location data was the following *Y* = μ + *P* + *e*. The means of significant different traits (*p* <0.05) among Ethiopian population was compared using the Fisher’s least significance difference test.

### GBS

Genomic DNA was isolated from seedlings of the 374 Ethiopian accessions based on the methods of Guillemaut, Marechal-Drouard [49]. DNA was re-purified using the ZR96 DNA Clean & Concentrator-5 (Zymo Research, Irvine, CA, USA). The GBS library was prepared and sequenced at the Institute for Genomic Diversity (University of Cornell, NY; http://www.biotech.cornell.edu/brc/genomic-diversity-facility) using the restriction enzyme *Ape*KI for digestion. The library had 384 unique barcodes including 4 random blanks (i.e., water). The GBS library was sequenced on four lanes (depth = 5.0) of an Illumina HiSeq 2500, and the GBS analysis pipeline (Tassel Version: 3.0.147), an extension of the Java program TASSEL [50], was used to call SNPs from the sequenced library by aligning tags to the BTx623 sorghum genome (*Sorghum bicolor* v.1.0; [51]). To ensure that sequencing error were not used in our analysis, we retained SNPs with <20% missing data and minor allele frequencies of >0.01 (i.e., SNPs present in at least three accessions) to obtain a final sample of 148,476 SNPs. The numbers of SNPs within genes and exons, and in the proximity of genes (5 kb up- and downstream), were determined using BEDTools [52].

### Population structure, LD, and effective population size

Population structure and admixed ancestry were assessed using a model-based clustering method implemented in ADMIXTURE [24]. Twenty-seven thousand three hundred and six unlinked SNPs (*r* ^2^ < 0.5) distributed across the genome were identified with PLINK [53] and used in the analysis. To determine the actual number of populations, ADMIXTURE was run with a 10-fold cross-validation procedure for K ranging from 1 to 20, and the K value with the lowest standard error was selected [24].

LD between SNPs for each chromosome was estimated in TASSEL 5.0 [50] using a sliding window of 25 bp. These estimates were based on SNPs with a minor allele frequency of >0.05 and *R* ^*2*^ between SNPs with a *p-*value of <0.001. Effective population size was determined based on LD values from SNPs with a minor allele frequency of >0.05 as implemented in SNeP [54].

### Fixation index and outlier detection

The pairwise fixation index among populations was estimated based on the method of Weir and Cockerham [55] using the R package Hierfstat [56]. To detect signatures of selection, *F*_ST_ outliers were detected among the 27,306 unlinked SNPs using BayeScan v.2.1 [28]. To reduce the identification of false positives, a 50,000-iteration burn-in period and thinning interval size of 10 were used, and the prior odds for the neutral model was up to 1,000. The prior odd threshold to identify *F*_ST_ outliers was determined using a false discovery rate of 0.001 as implemented in the “*plot_bayescan*” function in R.

### Cluster analysis

Pairwise genetic distances among the 374 Ethiopian accessions were calculated based on IBS as implemented in TASSEL 5.0 [50] using 27,306 previously identified unlinked SNPs. The resulting matrix was subjected to a clustering analysis using neighbor-joining and visualized using Interactive Tree of Life [57].

Genetic relationships among the NPGS Ethiopia collection and SAP were investigated by the identification of common SNPs in the SAP (265,487 SNPs [16]) and NPGS Ethiopia collection (148,475 SNPs). In total, 60,962 common SNPs were detected, and a subset of 26,026 unlinked (*r* ^2^ < 0.5) SNPs were identified in PLINK [53] for further analysis. Pairwise genetic distances among these 730 lines (374 and 356 from NPGS Ethiopia collection and SAP, respectively) were calculated based on IBS as implemented in TASSEL 5.0 [50], and the resulting matrix was subjected to a clustering analysis using neighbor-joining and visualized in Interactive Tree of Life [57].

### GWAS

The presence of population structure and familial relationships among accessions must be adequately accounted for in the GWAS model approach to reduce spurious associations [58]. The association analysis was performed with a compressed mixed linear model [59] implemented in the R package GAPIT [60] using 58,634 SNPs with a MAF > 0.5. Kinship was calculated as described by VanRaden [61], and a co-ancestry matrix from ADMIXTURE was included as a covariate in GAPIT to reduce spurious associations. Log Q–Q plots of *p-*values were examined to determine how well the models accounted for population structure and familial relatedness. The phenotypic data of PD, RA, and tannin content were normalized prior to the association analysis. Significant associations were determined for each trait using a false discovery rate-adjusted *p-*value of >0.05 [62] as implemented in GAPIT. Manhattan and Q–Q plots were visualized using the R package qqman [63].

## Additional files

Additional file 1: Figure S1. Linkage disequilibrium curves for the 10 sorghum chromosomes among 374 NPGS Ethiopian sorghum accessions using 58,634 SNPs with a MAF > 0.5. **Figure S2.** GWAS for tannins and phenols content using core set of 374 Ethiopian accessions from NPGS germplasm collection. (a) GWAS for tannin content. (b) GWAS for total phenol content. **Figure S3.** GWAS for plant height using a subset of 374 Ethiopian accessions from NPGS germplasm collection. **Figure S4.** QQ-plots from GWAS using a subset of 374 Ethiopian accessions from NPGS germplasm collection. (a) Tannin content, (b) Plant height using 374 accessions, (c) Plant height using 352 accessions, (d) Total phenol content, (e) Flowering using 352 accessions, (f) Fat content, (g) Protein content. (DOCX 638 kb)
Additional file 2: Table S1 GRIN data base information for 374 sorghum accessions from the U.S. National Plant Germplasm System genetic characterized by genotype-by-sequence analysis (GBS). **Table S2.** Coancestry coefficient matrix of 374 Ethiopian sorghum accessions from NPGS germplasm collection. **Table S3.** Phenotype analysis of 374 sorghum accessions from the U.S. National Plant Germplasm System evaluated at Isabela and Mayaguez, Puerto Rico in 2013 and 2014, respectively. **Table S4.** IBD genetic distance matrix for 374 Ethiopian accessions from NPGS using 27,306 SNPs. **Table S5.** Kinship Matrix for 374 Ethiopian accessions as decribed by VanRaden [61] using 58,634 SNPs. (XLSX 3336 kb)

## Abbreviations

3-DOA: 3-deoxyanthocyanidins
FL: Flowering time
*F*_ST_: Fixation index
G72: Average cold germination
GAPIT: Genome Association and Prediction Integrated Tool
GBS: Genotype-by-sequence
GWAS: Genome-wide association studies
IBS: Identity by state
ICRISAT: International Crops Research Institute for the Semi-arid Tropics
LD: Linkage disequilibrium
MAF: Minor allele frequency
NPGS: National plant germplasm system
PD: Panicle diameter
PH: Plant height
PL: Panicle length
Q–Q: Quantile–quantile
RA: Ratio length/diameter of panicle
SAP: Sorghum association panel
SNP: Single-nucleotide polymorphism
