# Supplementary material for: Genomic characterization of a core set of the USDA-NPGS Ethiopian sorghum germplasm collection: implications for germplasm conservation, evaluation, and utilization in crop improvement
Source: BMC Genomics. 2017 Jan 26;18:108. doi: 10.1186/s12864-016-3475-7 (PMC5270221; doi:10.1186/s12864-016-3475-7)
Supplement: Additional file 1: Figure S1. — Linkage disequilibrium curves for the 10 sorghum chromosomes among 374 NPGS Ethiopian sorghum accessions using 58,634 SNPs with a MAF > 0.5. Figure S2. GWAS for tannins and phenols content using core set of 374 Ethiopian accessions from NPGS germplasm collection. (a) GWAS for tannin content. (b) GWAS for total phenol content. Figure S3. GWAS for plant height using a subset of 374 Ethiopian accessions from NPGS germplasm collection. Figure S4. QQ-plots from GWAS using a subset of 374 Ethiopian accessions from NPGS germplasm collection. (a) Tannin content, (b) Plant height using 374 accessions, (c) Plant height using 352 accessions, (d) Total phenol content, (e) Flowering using 352 accessions, (f) Fat content, (g) Protein content. (DOCX 638 kb) [file 12864_2016_3475_MOESM1_ESM.docx]

**Figure 1 Supplementary** Linkage disequilibrium curves for the 10 sorghum chromosomes among 374 NPGS Ethiopian sorghum accessions using 58,634 SNPs with a MAF > 0.5.

**
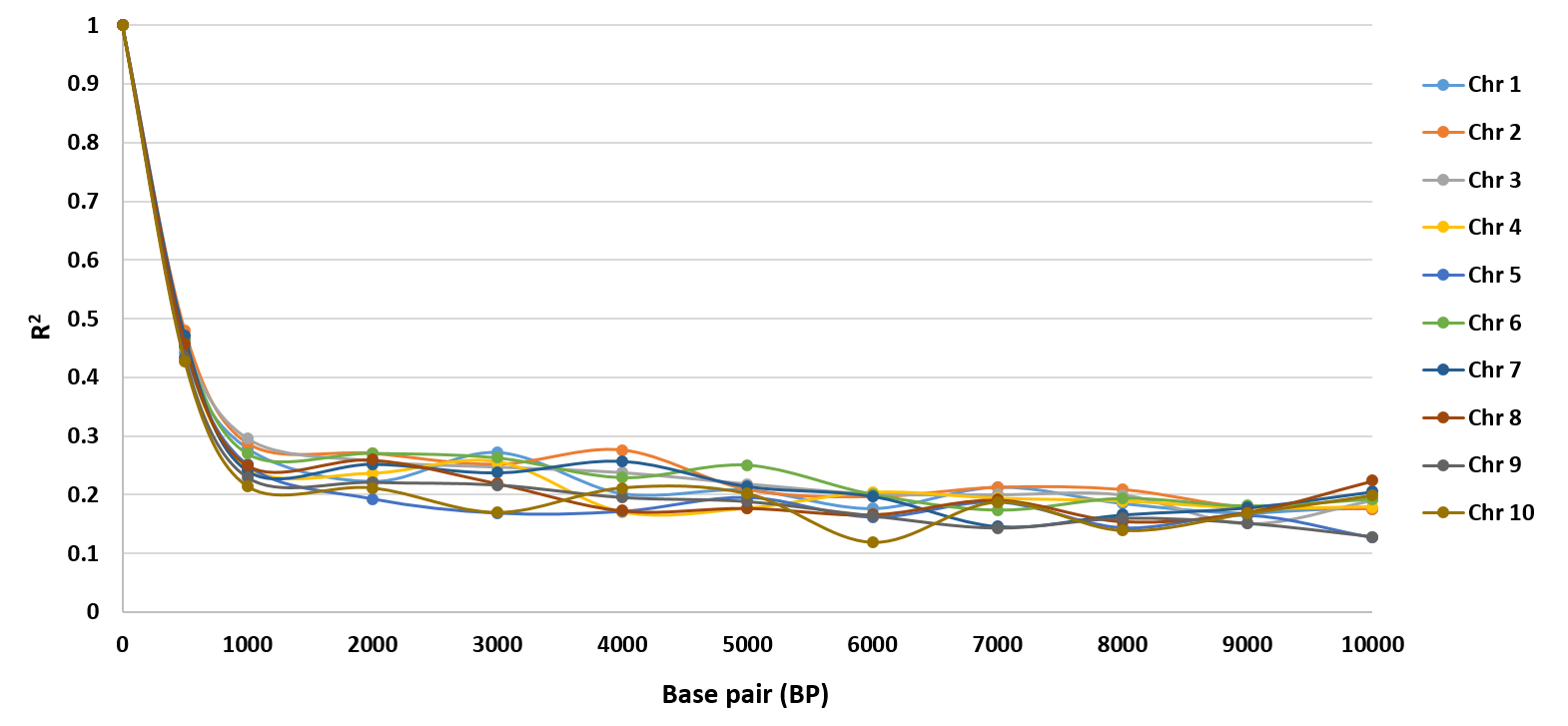
**

**Figure 2 Supplementary**. GWAS for tannins and phenols content using core set of 374 Ethiopian accessions from NPGS germplasm collection. **(a)** GWAS for tannin content. **(b)** GWAS for total phenol content


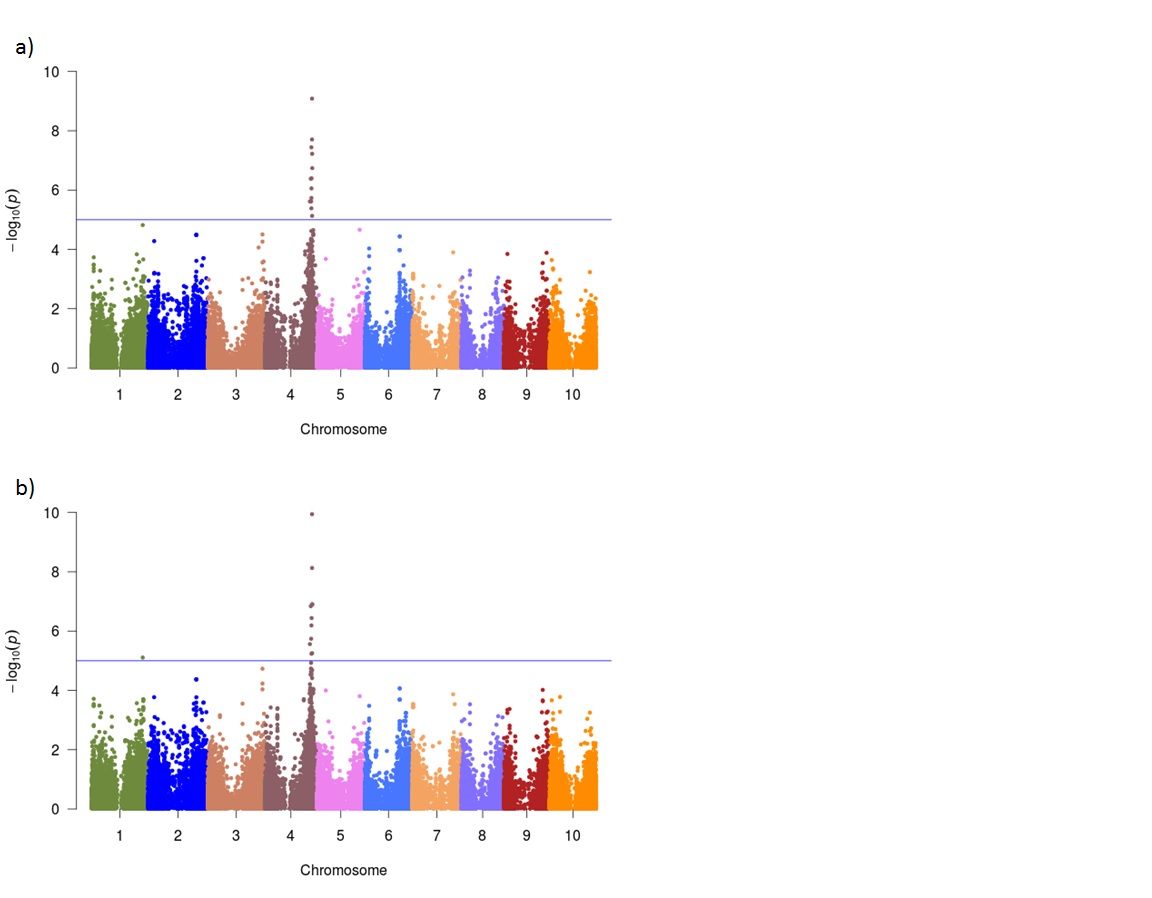


*Tan1*


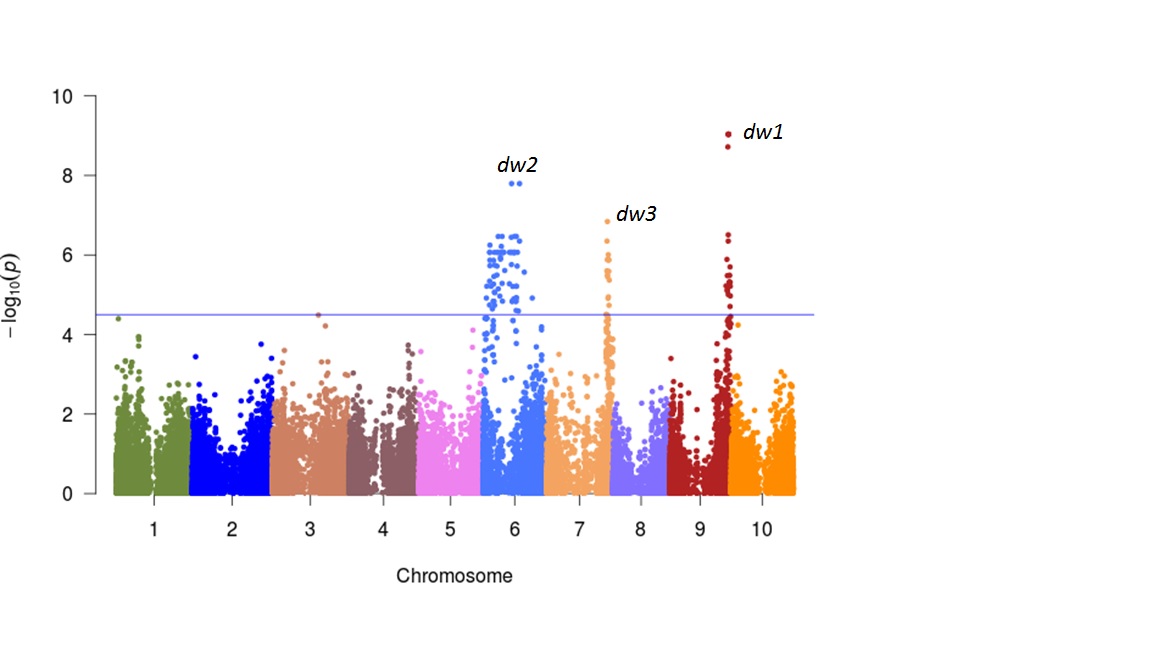
**Figure 3 Supplementary**. GWAS for plant height using a subset of 374 Ethiopian accessions from NPGS germplasm collection

**
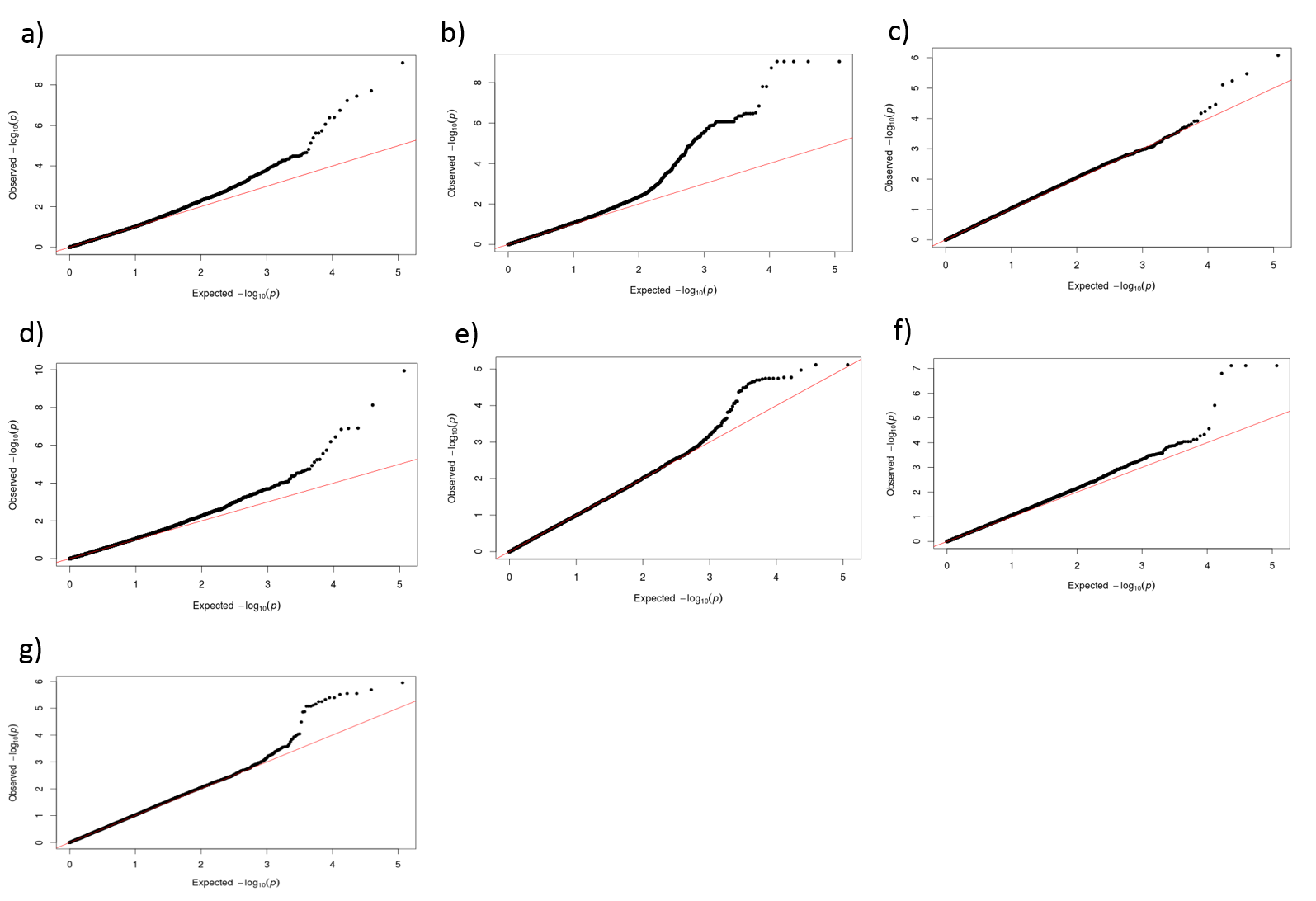
Figure 4 Supplementary** QQ-plots from GWAS using a subset of 374 Ethiopian accessions from NPGS germplasm collection. **(a)** Tannin content, **(b)** Plant height using 374 accessions, **(c)** Plant height using 352 accessions, **(d)** Total phenol content, **(e)** Flowering using 352 accessions, **(f)** Fat content, **(g)** Protein content.
